# Apelin is expressed throughout the human kidney, is elevated in chronic kidney disease & associates independently with decline in kidney function

**DOI:** 10.1111/bcp.15446

**Published:** 2022-07-19

**Authors:** Duuamene Nyimanu, Fiona A. Chapman, Peter J. Gallacher, Rhoda E. Kuc, Thomas L. Williams, David E. Newby, Janet J. Maguire, Anthony P. Davenport, Neeraj Dhaun

**Affiliations:** ^1^ Division of Experimental Medicine and Immunotherapeutics University of Cambridge Cambridge UK; ^2^ Centre for Cardiovascular Science, The Queen's Medical Research Institute University of Edinburgh/British Heart Foundation Centre of Research Excellence Edinburgh UK; ^3^ Department of Renal Medicine Royal Infirmary of Edinburgh Edinburgh UK

**Keywords:** apelin, cardiovascular system, chronic kidney disease

## Abstract

**Aims:**

Chronic kidney disease (CKD) is common and cardiovascular disease (CVD) is its commonest complication. The apelin system is a potential therapeutic target for CVD but data relating to apelin in CKD are limited. We examined expression of the apelin system in human kidney, and investigated apelin and Elabela/Toddler (ELA), the endogenous ligands for the apelin receptor, in patients with CKD.

**Methods:**

Using autoradiography, immunohistochemistry and enzyme‐linked immunosorbent assay, we assessed expression of apelin, ELA and the apelin receptor in healthy human kidney, and measured plasma apelin and ELA in 155 subjects (128 patients with CKD, 27 matched controls) followed up for 5 years. Cardiovascular assessments included blood pressure, arterial stiffness (pulse wave velocity) and brachial artery flow‐mediated dilation. Surrogate markers of endothelial function (plasma asymmetric dimethylarginine and endothelin‐1) and inflammation (C‐reactive protein and interleukin‐6) were measured.

**Results:**

The apelin system was expressed in healthy human kidney, throughout the nephron. Plasma apelin concentrations were 60% higher in women than men (6.48 [3.62–9.89] *vs*. 3.95 [2.02–5.85] pg/mL; *P* < .0001), and increased as glomerular filtration rate declined (*R* = −0.41, *P* < .0001), and albuminuria rose (*R* = 0.52, *P* < .0001). Plasma apelin and ELA were associated with vascular dysfunction. Plasma apelin associated independently with a 50% decline in glomerular filtration rate at 5 years.

**Conclusion:**

We show for the first time that the apelin system is expressed in healthy human kidney. Plasma apelin is elevated in CKD and may be a potential biomarker of risk of decline in kidney function. Clinical studies exploring the therapeutic potential of apelin agonism in CKD are warranted.

**What is already known about this subject**

- Within the cardiovascular system, apelin lowers blood pressure and increases cardiac inotropy.
- Patients with chronic kidney disease (CKD) commonly have hypertension, and cardiovascular disease is their commonest complication. There are few data on apelin in healthy human kidney or in CKD.
- We aimed to characterize the expression of the apelin system throughout the human kidney and examined plasma apelin and ELA concentrations in patients with CKD, to advance knowlegde of the system in humans and explore the impact of CKD.

**What this study adds**

- We have shown for the first time that the apelin system is expressed throughout the human kidney.
- In patients with CKD, plasma apelin concentration increases as kidney function declines. Plasma apelin is associated with risk of future decline in kidney function.
- Studies of the effect of apelin in humans with kidney disease are now justified.

## 1 INTRODUCTION

Chronic kidney disease (CKD) is increasingly common with a current global prevalence of ~10%.^1^ It encompasses a heterogenous group of conditions that result in reduced kidney function and proteinuria, with the eventual development of end‐stage kidney disease (ESKD). CKD is independently associated with incident cardiovascular disease.^2^ Cardiovascular risk increases as estimated glomerular filtration rate (eGFR) declines, and is greatest in those with ESKD. Current treatment guidelines for CKD focus on reducing blood pressure (BP) and proteinuria using inhibitors of the renin–angiotensin–aldosterone system (RAAS). Recent studies have also identified the potential therapeutic benefits in CKD of sodium glucose cotransporter 2 (SGLT2) inhibitors^3^ and glucagon‐like peptide 1 receptor agonists.^4^ Whilst these developments are encouraging, there remains an unmet need for newer therapies for patients with CKD that might delay the progression to ESKD and offer broader cardiovascular protection.

Apelin is an endogenous peptide and the ligand for the apelin receptor (previously known as APJ). Both apelin and its receptor are widely expressed throughout the vasculature, but especially on the endothelium and vascular smooth muscle cells.^5^ Apelin metabolism has not been fully defined. Angiotensin converting enzyme II, plasma kallikrein, furin and neprilysin are recognised to cleave apelin peptide but only neprilysin has been shown to fully inactivate it.^6^, ^7^, ^8^, ^9^, ^10^ Preclinical and clinical studies have shown that apelin is a potent inotrope and an endothelium‐dependent vasodilator that lowers BP,^11^, ^12^, ^13^ with actions opposing those of angiotensin II.^14^, ^15^, ^16^, ^17^ Although experiments in rats have demonstrated apelin mRNA in glomeruli and inner medulla,^18^ there are no data in healthy human kidney. Elabela/Toddler (ELA) is a second ligand for the apelin receptor and is critical for cardiovascular development.^19^ In adult humans, ELA localises to the kidney and vascular endothelium.^20^, ^21^

Both apelin and ELA have been suggested as being important in reducing inflammation within the kidney and in promoting diuresis.^22^, ^23^, ^24^, ^25^, ^26^ In a model of septic shock, both peptides improve survival although ELA was of greatest benefit, improving cardiac function and limiting renal injury.^27^ Given these potentially beneficial cardiovascular and renal effects, the apelin system is an appealing novel therapeutic target for kidney disease. Here, we characterized the expression of the apelin system throughout the human kidney and examined plasma apelin and ELA concentrations in patients with CKD.

## 2 METHODS

Using autoradiography, immunohistochemistry and enzyme‐linked immunosorbent assay, we assessed expression of apelin, ELA and the apelin receptor throughout the normal human kidney. Next, we recruited patients with nondiabetic CKD from the general renal outpatient clinic at the Royal Infirmary of Edinburgh and measured and plasma apelin and ELA. Patients were excluded if they had known cardiovascular disease (ischaemic heart disease, congestive cardiac failure or stroke) to avoid a confounding effect on the apelin system. Patients were followed‐up for 5 years. Baseline cardiovascular assessments included BP, arterial stiffness and brachial artery flow‐mediated dilation. Plasma asymmetric dimethylarginine (ADMA) and endothelin‐1 (ET‐1) were used as measures of endothelial function, and C‐reactive protein (CRP) and interleukin‐6 (IL‐6) as measures of inflammation.

### 2.1 Nomenclature of targets and ligands

Key protein targets and ligands in this article are hyperlinked to corresponding entries in http://www.guidetopharmacology.org, and are permanently archived in the Concise Guide to PHARMACOLOGY 2021/22.^28^

## 3 RESULTS

### 3.1 Expression of the apelin system in human kidney

**FIGURE 1:**
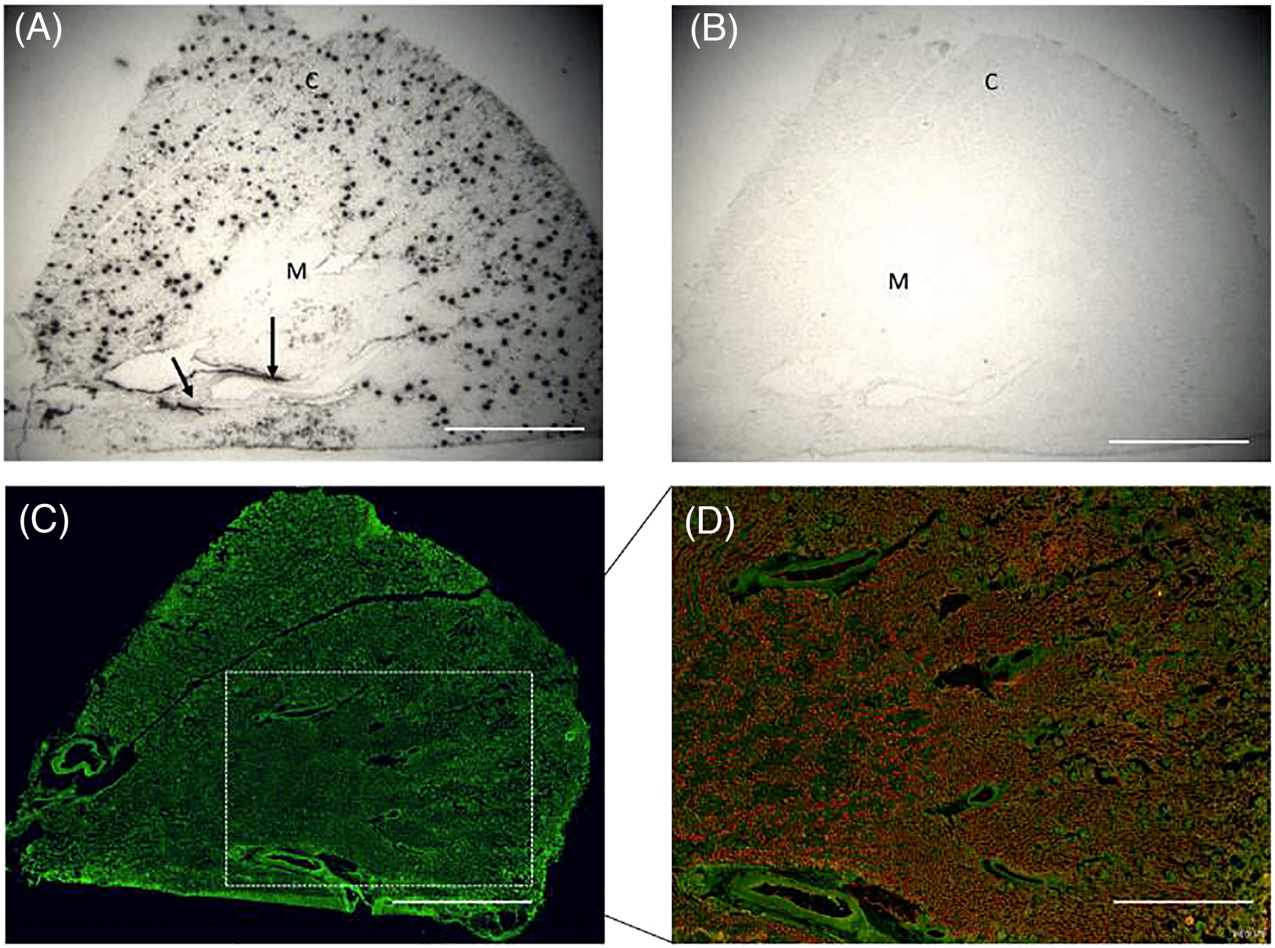
The distribution of the apelin receptor within human kidney indicated by [^125^I]apelin‐13 binding sites with each black dot representing of binding to apelin receptors in the glomerulus. The arrow indicates binding within a blood vessel (A). Nonspecific binding is seen in the presence of [Pyr^1^]apelin‐13 (B). Apelin receptor‐like immunoreactivity in human kidney (green; C); Merged, aquaporin 1 (red; D). C: renal cortex; M: renal medulla. Scale bar = 5 mm

Apelin (*APLN*), ELA (*APELA*) and apelin receptor (*APLNR*) mRNA were expressed to a similar extent in human kidney cortex and medulla. Using enzyme‐linked immunosorbent assay, the concentration of apelin and ELA peptides in the kidney were 0.45 ± 0.17 and 1.24 ± 0.29 ng/mL, respectively (Figure S1). In whole kidney sections, autoradiography demonstrated dense apelin receptor binding throughout the cortex, and to a lesser degree in the medulla, with high densities discretely localized to the glomerulus. In the presence of [Pyr^1^]apelin‐13, binding of radiolabelled apelin‐13 was entirely abolished (Figure 1A,B). A similar distribution of apelin receptor was detected by immunohistochemistry (Figure 1C,D). In saturation binding experiments [Pyr^1^]apelin‐13 bound with a single, subnanomolar affinity (K_D_ 0.34 ± 013 nM, Hill slope 1.04 ± 0.05) with receptor density of 15.41 ± 2.29 fmol/mg.

#### 3.1.1 Renal vasculature & glomerulus

**FIGURE 2:**
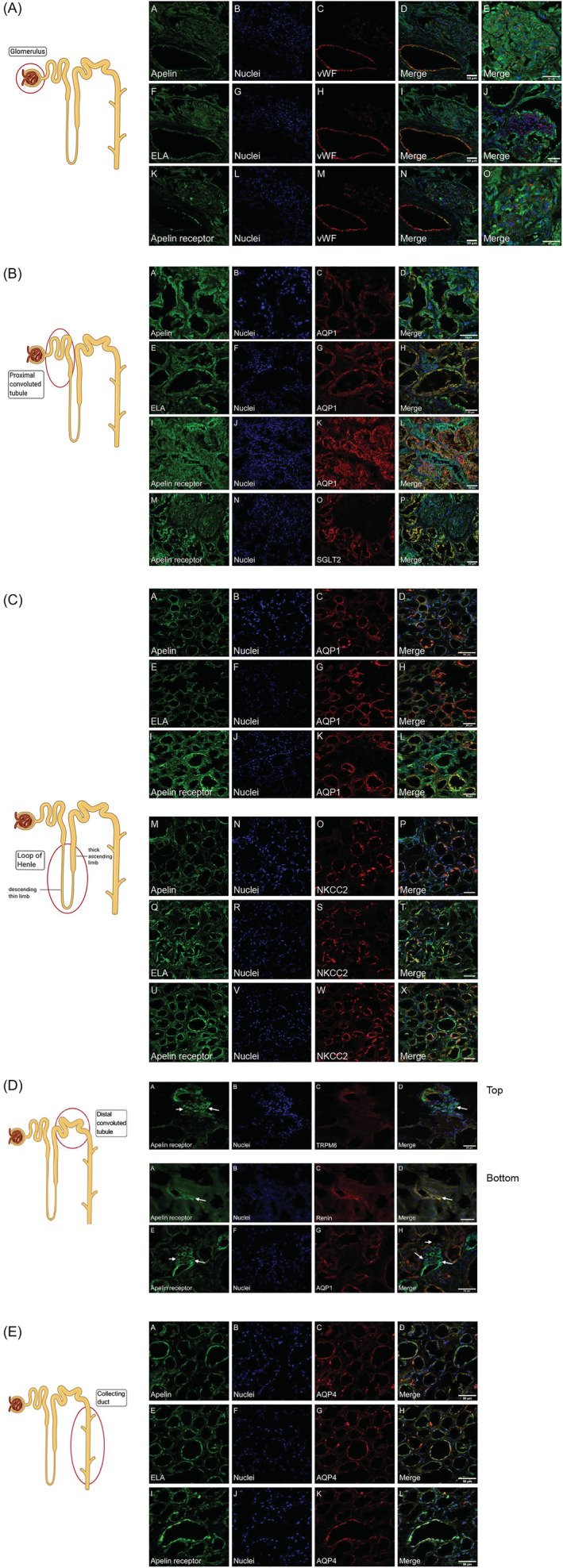
(A) Localization of apelin (A–E), ELA (F–J) and apelin receptor (K–O) protein to renal blood vessel (A, F, K) and glomerulus (E, J, O). Whilst there is colocalization of all proteins with von Willebrand Factor (vWF, an endothelial cell marker) within the vessel wall, there does not appear to be colocalization of apelin, ELA or apelin receptor proteins with the endothelial marker within the glomerulus. Scale bar = 50 μm. (B) Localization of apelin (A–D), ELA (E–H) and apelin receptor (I–L) protein in the proximal convoluted tubule (PCT) within the cortex. Aquaporin 1 (AQP1; C, G, K) is expressed by proximal tubular epithelial cells and acts as a marker of the PCT. Apelin receptor (M–P) protein also colocalizes with the sodium–glucose cotransporter 2 (SGLT2; O) protein within the PCT. Scale bar = 50 μm. (C) Localization of apelin (A–D), ELA (E–H) and apelin receptor (I–L) protein to the descending thin limb of the Loop of Henle indicated by expression of aquaporin 1 (AQP1; C, G, K). Apelin (M–P), ELA (Q–T) and apelin receptor (U–X) protein localize to the thick ascending limb of the loop of Henle within the renal cortex, indicated by the expression of the sodium–potassium–chloride cotransporter (NKCC2; O, S, W). Scale bar = 50 μm. (D) Top panel: apelin receptor expression in the distal convoluted tubule (DCT) indicated by colocalization with the magnesium channel transient receptor potential cation channel subfamily M member 6 (TRPM6; A–D). Arrow indicates apelin receptor expression to cells within the juxtaglomerular apparatus (D). Bottom panel: apelin receptor protein colocalises with renin (A–D) within the juxtaglomerular apparatus (D). Aquaporin 1 (AQP1) indicates proximal convoluted tubule adjacent to the juxtaglomerular apparatus. Scale bar = 50 μm. (E) Apelin (A–D), ELA (E–H) and the apelin receptor (I–L) are found within the collecting duct. Aquaporin 4 (AQP4) localizes to the principal cells of the collecting duct. Scale bar = 50 μm.

In all sections examined, apelin, ELA and apelin receptor protein colocalized with von Willebrand factor, an endothelial cell marker, in both intrarenal arteries and renal arterioles. Low‐level apelin receptor immunoreactivity was also found in vascular smooth muscle cells (Figure S2). Throughout the nephron, apelin, ELA and apelin receptor protein localized to epithelial cells with punctate staining of all 3 proteins observed within the glomerulus although, interestingly, there was very little localization to the endothelium here suggesting expression by other cell types such as podocytes (Figure 2A).

#### 3.1.2 Proximal convoluted tubule

Immunofluorescence staining for apelin, ELA and apelin receptor was seen in epithelial cells of the proximal convoluted tubule (PCT), identified by colocalization with cortical aquaporin‐1 (AQP1; Figure 2B). Whereas apelin immunoreactivity was seen at the apical membrane, ELA and apelin receptor protein were present on both the apical and basolateral membranes of the epithelial cells (Figure S3). Interestingly, we observed colocalization of the apelin, ELA and apelin receptor with the SGLT2, suggesting that apelin may play a role in glucose handling within the kidney (Figure 2B).

#### 3.1.3 Loop of Henle

Next, we examined the thin descending and thick ascending limbs of the loop of Henlé, defined by epithelial expression of medullary AQP1 and the sodium–potassium–chloride cotransporter (NKCC2), respectively. Apelin, ELA and the apelin receptor were expressed throughout the loop of Henlé (Figure 2C).

#### 3.1.4 Distal convoluted tubule

We observed apelin receptor immunoreactivity colocalizing with the magnesium channel transient receptor potential cation channel subfamily M member 6 (TRPM6), a specific marker of epithelial cells in the distal convoluted tubule (Figure 2D).

#### 3.1.5 Juxtaglomerular apparatus

Of significance, the juxtaglomerular apparatus, found in close proximity to the PCT and distal convoluted tubule, also demonstrated staining for apelin receptor as marked by colocalization with renin (Figure 2D).

#### 3.1.6 Collecting duct

Finally, apelin, ELA and apelin receptor protein were all present within epithelial cells of the collecting duct, identified by colocalization with the water channel, aquaporin 4 (AQP4) in the principal cells (Figure 2E).

### 3.2 Plasma apelin and ELA in healthy subjects and patients with CKD

Having demonstrated expression of the apelin system throughout the human kidney, next we measured apelin and ELA concentrations in patients with nondiabetic CKD (*n =* 128) and age‐ and sex‐matched healthy volunteers (*n =* 27; Table 1).

**TABLE 1:** Baseline characteristics of participants. Data are mean ± SEM

	Participants	
	HV, *n = 27*	CKD, *n = 128*
Age, y	47 ± 2	46 ± 1
Sex (%)		
Male	15 (56)	80 (63)
Female	12 (44)	48 (37)
**Primary renal disease (%)**		
Glomerulonephritis		66 (51)
Unknown		22 (17)
Polycystic kidney disease		18 (14)
Reflux nephropathy		10 (8)
Other		5 (4)
Renal stone		2 (1.5)
Thin basement membrane disease		2 (1.5)
Obstruction		1 (1)
Medullary sponge kidney		1 (1)
Congenital cysteinuria		1 (1)
Serum creatinine, μmol/L^a^	77.6 ± 2.6	150.0 ± 9.0
CKD stage (%)		
Stage 1		33 (26)
Stage 2		33 (26)
Stage 3		39 (30)
Stage 4		18 (14)
Stage 5		5 (4)
Albumin:creatinine ratio, mg/g		106.4 ± 14.8
Systolic blood pressure, mmHg	111 ± 3	120 ± 1
Diastolic blood pressure, mmHg	70 ± 2	74 ± 1
Mean arterial pressure, mmHg	84 ± 2	89 ± 1
**Medications (%)**		
Alpha blocker		12 (9)
ACE inhibitor		78 (61)
ARB		26 (20)
Beta blocker		32 (25)
Calcium channel blocker		34 (27)
Diuretic		20 (16)
Statin		45 (35)

ACE, angiotensin converting enzyme; ARB, angiotensin receptor blocker; HV, healthy volunteer; CKD, chronic kidney disease.

a To convert creatinine from μmol/L to mg/dL, divide by 88.4.

#### 3.2.1 Associations with baseline characteristics

Plasma apelin concentration did not associate with age. Intriguingly, it was ~60% higher in women (*n =* 60) than men (*n =* 95; 6.48 [3.62–9.89] *vs*. 3.95 [2.02–5.85] pg/mL, *P* < .0001). No differences were seen in participant characteristics between men and women in our study population (including primary renal disease, CKD stage or prescribed medications). Plasma ELA concentration associated weakly with plasma apelin concentration (*R =* .23, *P* < .05), although there was no association with age or sex (Figure S4).

**FIGURE 3:**
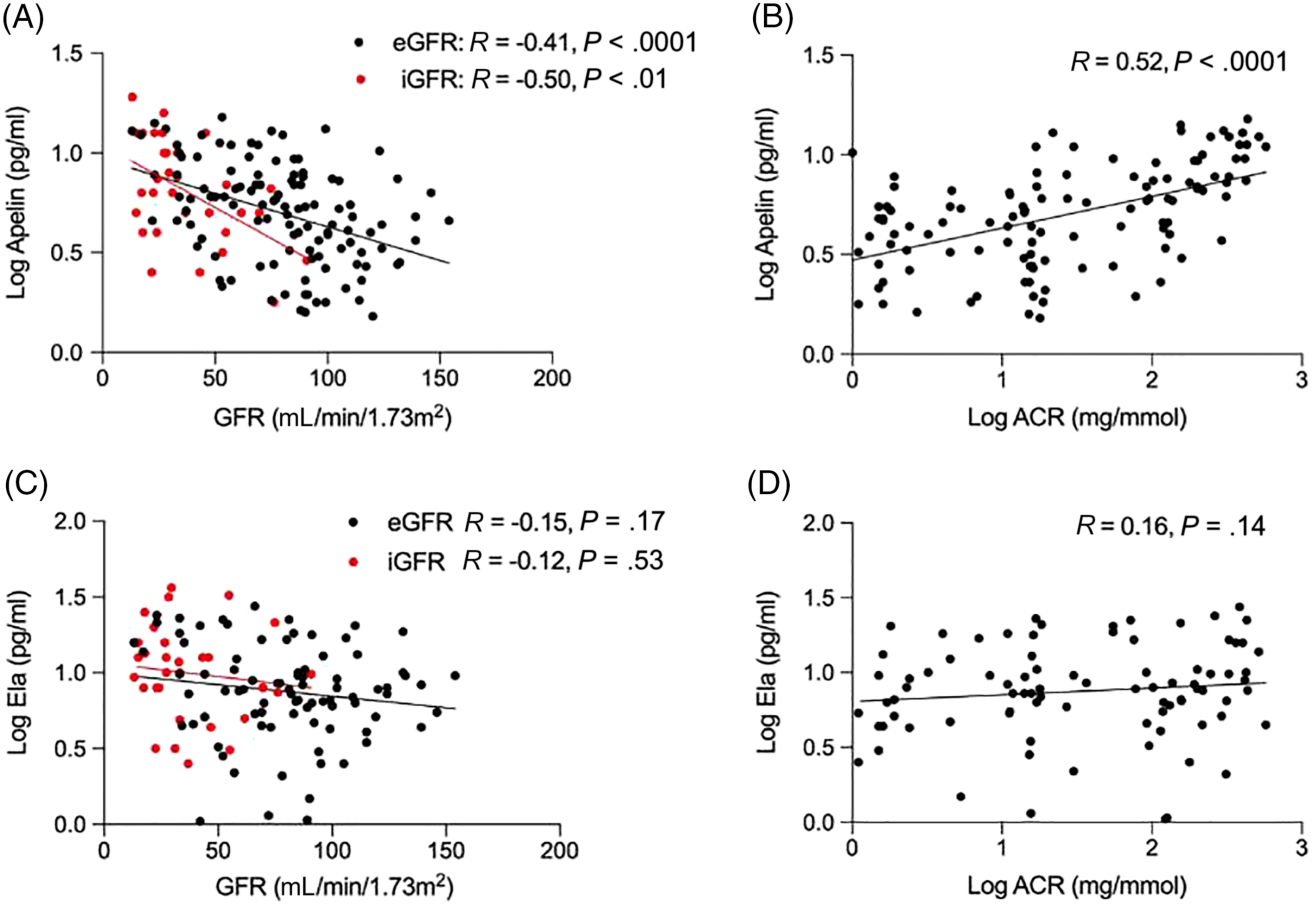
Associations of apelin with estimated (eGFR) and inulin‐measured (iGFR) glomerular filtration rate (A), and albuminuria (ACR: albumin: creatinine ratio; B); corresponding associations of ELA with eGFR and iGFR (C) and albuminuria (D). We included 128 patients with CKD and 27 healthy volunteers

Apelin correlated inversely with both eGFR and inulin‐measured actual glomerular filtration rate (iGFR) and was ~1.6‐fold higher in those with an eGFR <60 mL/min/1.73 m^2^ compared to those with an eGFR >60 mL/min/1.73 m^2^ (5.59 [3.56–9.89] *vs*. 3.52 [1.92–5.97] pg/mL, *P* < .001). Additionally, plasma apelin concentrations rose as urinary albumin excretion increased and fell as urinary sodium excretion increased (*R =* −.50, *P* < .01). A similar trend was seen between plasma ELA concentration and eGFR, iGFR, albuminuria or sodium excretion (Figure 3).

#### 3.2.2 Associations with vascular function and inflammation

**FIGURE 4:**
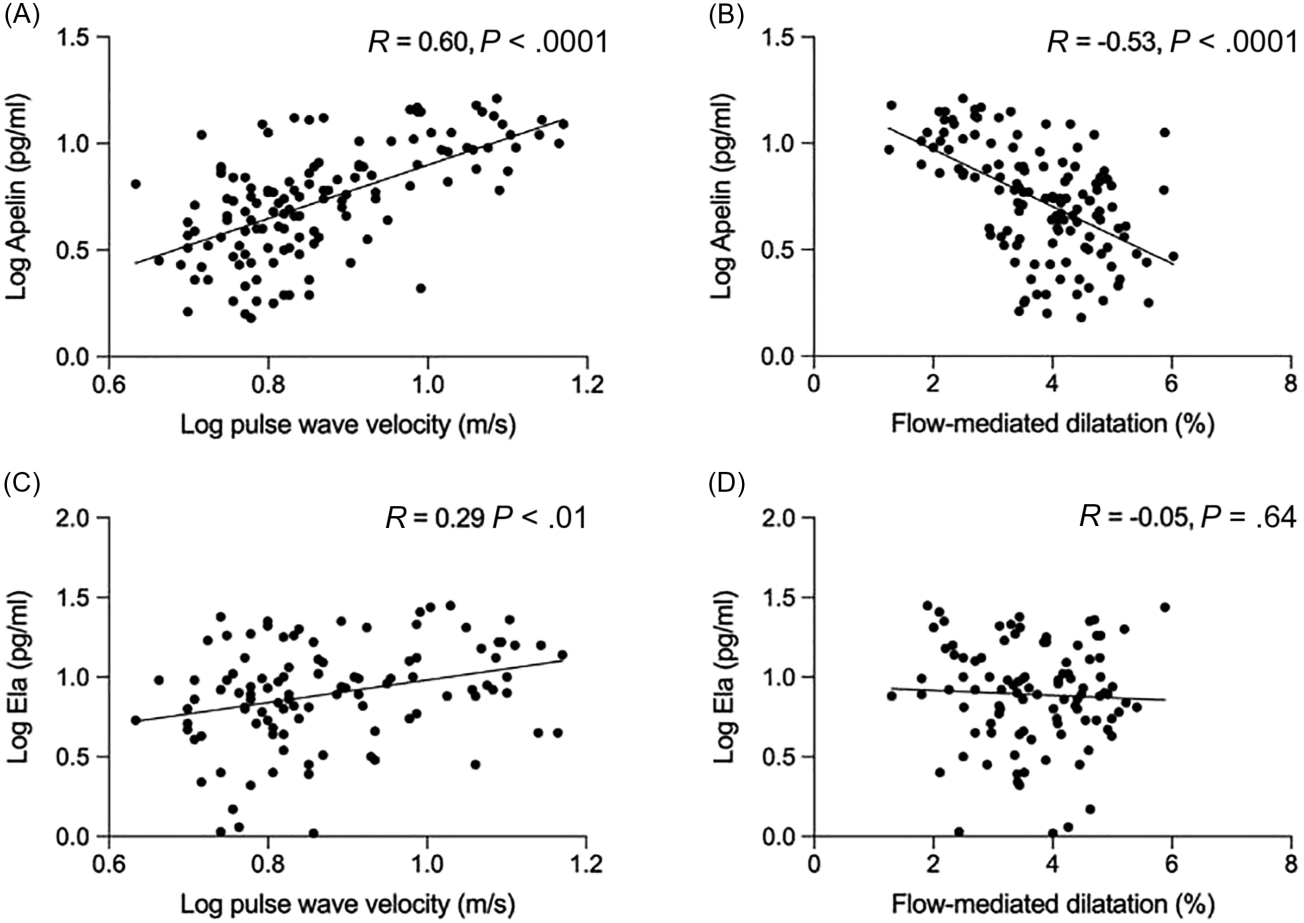
Associations of apelin with arterial stiffness (A) and endothelial function (B), and corresponding associations of ELA (Ela) with arterial stiffness (C) and endothelial function (D). We included 128 patients with CKD and 27 healthy volunteers

Systolic BP correlated weakly with plasma apelin (*R =* .22, *P* < .01) but not with plasma ELA concentrations (*R =* .08, *P* = .40). Additionally, there was a positive correlation between apelin and arterial stiffness, measured by pulse wave velocity, and endothelial dysfunction, measured by flow‐mediated dilation, with arterial stiffness also significantly correlated with plasma ELA concentrations (Figure 4).

**FIGURE 5:**
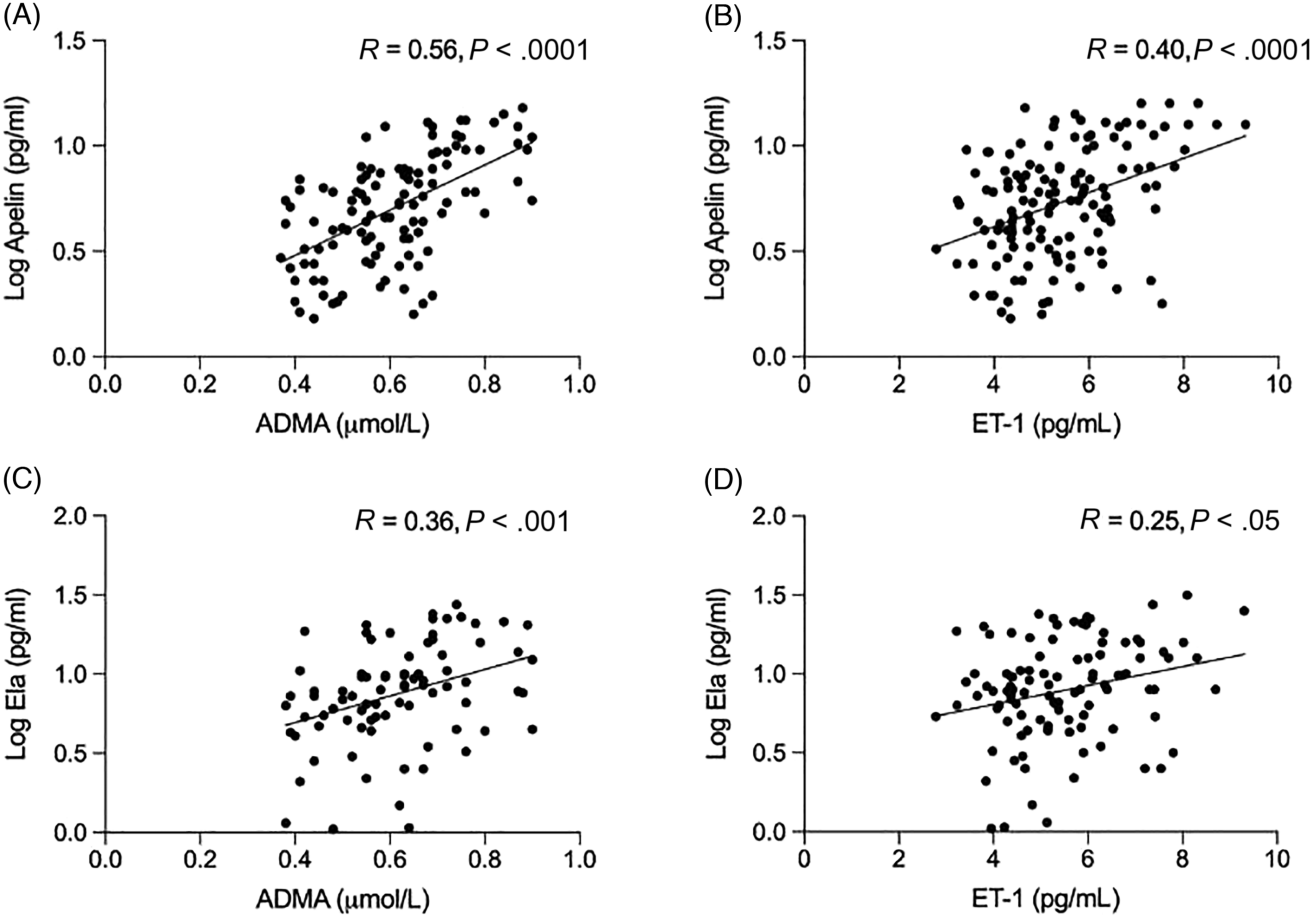
Associations of apelin concentration with asymmetric dimethylarginine (ADMA; A) and endothelin‐1 (ET‐1; B), and corresponding associations of ELA (Ela) with ADMA (C) and ET‐1 (D). We included 128 patients with CKD and 27 healthy volunteers

Both plasma apelin and ELA concentrations correlated with plasma concentrations of ADMA, an endogenous inhibitor of nitric oxide synthesis, and plasma ET‐1, a potent endogenous vasoconstrictor (Figure 5). The concentration of big ET‐1, the precursor peptide of ET‐1, was also associated with plasma apelin and ELA concentrations (*R =* .29, *P* < .01 and *R =* .25, *P* < .05, respectively). Similarly, the ratio of big ET‐1:ET‐1, which rises as ET‐1 production goes up,^29^, ^30^ increased in line with plasma apelin and ELA concentrations, and falling eGFR (Figure S5).

Apelin did not associate with measures of inflammation (IL‐6, *R =* .06, *P* = .60; high‐sensitivity CRP, *R =* −.03, *P* = .75), ELA correlated with IL‐6 (*R =* .28, *P* < .05) but not high‐sensitivity CRP (*R =* .03, *P* = .79).

In multivariable analysis, albuminuria, arterial stiffness and ADMA independently associated with plasma apelin concentration (Table 2). No factor was independently associated with plasma ELA concentration (Table S1).

**TABLE 2:** Multivariable analysis model identifying urinary albumin excretion, pulse wave velocity and serum asymmetric dimethylarginine to be independently associated with plasma apelin concentrations

	Beta coefficient	Standard error	*t* statistic	*p* value
Sex	0.14	0.13	1.03	.31
GFR	−0.004	0.002	−1.65	.10
Log ACR	0.085	0.03	2.82	**<.01**
Log PWV	0.56	0.25	2.26	**<.05**
FMD	−0.13	0.07	−1.74	.08
ET‐1	0.02	0.06	0.33	.74
ADMA	1.27	0.57	2.23	**<.05**

ACR, albumin: creatinine ratio; ADMA, asymmetric dimethylarginine; ET‐1, endothelin‐1; FMD, flow‐mediated dilatation; GFR, glomerular filtration rate; PWV, pulse wave velocity.

#### 3.2.3 Apelin is independently associated with eGFR decline

Finally, we assessed factors associated with eGFR decline. Follow‐up data at 5 years were available for 136 participants. Univariate logistic regression found that sex, baseline plasma apelin concentration, albuminuria and eGFR were associated with a ≥50% fall in eGFR at 5 years, a fall in eGFR that occurred in 49 participants (36%). In multivariable logistic regression, only baseline plasma apelin associated significantly with eGFR decline (Table 3). In our cohort of patients with CKD, 24/128 patients reached ESKD within 5 years. Baseline plasma apelin was higher in patients reaching ESKD than in those that did not (10.10 [7.83–13.0] *vs*. 4.00 [2.30–5.99] pg/mL, *P* < .0001).

**TABLE 3:** Multivariate logistic regression showing an independent association between plasma apelin level and a ≥50% fall in estimated GFR at 5 years

	Regression coefficient	Odds ratio (95% CI)	*P* value
Apelin (pg/mL)	2.181	8.859 (3.199–61.52)	**<.01**
ACR (mg/mmol)	−0.001	1.000 (0.991–1.009)	.91
Baseline GFR (ml/min/1.73m^2^)	0.015	1.015 (0.980–1.054)	.39
Sex (male)	−2.217	0.109 (0.006–0.974)	.074

r^2^ of model = 0.83.

ACR, albumin: creatinine ratio; CI, confidence interval; GFR, glomerular filtration rate.

## 4 DISCUSSION

For the first time, we have characterized the expression of the apelin system in human kidney and investigated plasma apelin and ELA concentrations in patients with CKD. We have shown that the apelin system is present in renal vasculature and throughout the nephron. Our clinical data demonstrate that women have a higher plasma apelin concentration than men and that there are significant relationships between plasma apelin and kidney function, as well as between apelin and ELA and independent surrogate measures of cardiovascular risk. Importantly, circulating apelin is independently associated with eGFR decline. Our findings are important not only in advancing our understanding of the apelin system in kidney disease, but also when considering apelin as a potential therapy for these patients.

We show similar expression of apelin, ELA and the apelin receptor in kidney cortex and medulla but whereas apelin localizes with renal vasculature outside the glomerulus, this is less clear within the glomerulus. Consistent with presence of apelin receptor to nonendothelial cells within the glomerulus, receptor protein has been shown to colocalize with nephrin and synaptopodin in cultured mouse podocytes and glomeruli in mouse kidney.^31^ It remains to be determined what the effects of apelin are on these multifunctional cells. Intriguingly, we show for the first time the presence of the apelin receptor within the juxtaglomerular apparatus. Here, activation of the RAAS occurs when low tubular sodium chloride is detected by the macula densa. There is recognized cross‐talk between the apelin system and the RAAS. Our data might suggest that apelin acts within the juxtaglomerular apparatus to regulate RAAS activation with subsequent effects on BP and glomerular filtration.^32^ This is supported by the colocalization of the apelin receptor with NKCC2 within the loop of Henlé. The transport of sodium and chloride by this cotransporter at the macula densa acts as the first step in tubuloglomerular feedback in signalling renin response.^33^

The colocalization of the apelin receptor and the SGLT2 within the PCT is particularly interesting. In addition to its cardiovascular benefits, apelin is recognized to influence glucose homeostasis and can increase insulin sensitivity.^34^ SGLT2 inhibitors have recently been licensed for the treatment of type 2 diabetes mellitus. They act by inhibiting the SGLT2 channel in the PCT, promoting glycosuria and improving glycaemic control. However, they also reduce BP and major cardiovascular endpoints in these patients and have recently been shown to have beneficial renal outcomes in patients with diabetic and nondiabetic CKD.^3^ There is recognized coexpression of the apelin receptor and the angiotensin II‐type 1 receptor and the vasopressin 2 receptor, and functional interaction of these systems.^32^, ^35^, ^36^ It would be particularly interesting to explore whether there may be similar interactions between the apelin receptor and SGLT2 within the PCT. Future studies might explore potential synergy in targeting both systems therapeutically in kidney disease. We hypothesize that apelin might act on the SGLT2 pathway to promote glucose excretion from the kidney and therefore have a beneficial effect in CKD patients.

Our clinical data show that plasma apelin concentrations increase as kidney function declines. This might reflect increased vascular production or reduced renal clearance. We observed the same trend for ELA. Previous studies that have explored plasma apelin concentrations in patients with CKD have found both lower and higher concentrations than in health.^37^, ^38^, ^39^ Notably, these studies included patients with comorbidities (coronary artery disease, diabetes, heart failure) that are recognized to affect the apelin system.^34^, ^40^ Thus, we specifically excluded patients with diabetes or overt cardiovascular disease from our study. Additionally, these earlier studies had significant heterogeneity amongst patients with CKD and control subjects, and measured apelin using different assays. Key strengths of our study include the large study population, inclusion of patients with CKD without confounding comorbidity, a well‐matched group of healthy volunteers, and use of an assay that reliably measures plasma apelin isoforms, the most abundant of which is [Pyr^1^]apelin‐13.^41^

Optimizing salt and water excretion and reducing albuminuria (a strong independent predictor of cardiovascular and renal risk) are key strategies in the management of patients with CKD as both improve outcomes.^42^ Our data show that apelin associates with both. Indeed, albuminuria was an independent predictor of plasma apelin concentrations. Within the kidney, apelin expression localizes to the vasculature, and apelin has a clear role in fluid balance, with recognized crosstalk with vasopressin.^18^, ^22^ In models, apelin acts directly on the tubule to promote diuresis.^22^ There are currently no clinical studies exploring the effects of apelin on renal salt and water handling. Such studies might lead to a novel treatment for CKD and inform clinical trials of apelin in cardiovascular disease, where an inability to excrete salt and water appropriately is a prominent feature.

Our finding that women have higher plasma apelin concentrations than men is novel and is not accounted for by a GFR difference between the 2 groups. Sex differences are well established for other peptides involved in BP control such as angiotensin (1‐7) and ET‐1.^43^, ^44^ It is noteworthy that the apelin gene is located on the X chromosome, and there are sex‐specific genetic polymorphisms of both apelin and its receptor that confer differing risks of hypertension.^45^ Relevant to our findings of sex differences in plasma apelin concentration is the fact that there is reciprocal regulation between the RAAS and apelin system. In a rodent model of heart failure, infusion of angiotensin II downregulated cardiac apelin expression and this was prevented by blockade of the angiotensin II type 1 receptor.^15^ Oestrogens reduce angiotensin II type 1 receptor density in animal models and so it is plausible that this could increase apelin expression and plasma apelin concentrations.^46^ It is recognized that women with nondiabetic CKD progress more slowly to ESKD than men.^47^ The reasons for this are unclear but sex‐specific alterations in cardiovascular hormones may contribute.

CKD is characterized by arterial stiffening, vascular dysfunction, inflammation and accelerated atherosclerosis.^48^, ^49^ Here, we show that apelin associates independently with arterial stiffness and endothelial dysfunction, both surrogate measures of cardiovascular risk.^50^, ^51^ In keeping with these data, plasma apelin and ELA concentrations correlated positively with ET‐1 and ADMA, reflecting an imbalance towards vasoconstriction with more ET‐1 and less nitric oxide, respectively. Apelin reduces ET‐1‐induced vasoconstriction^11^ and so it is plausible that there is a compensatory increase in apelin production to counteract ET‐1 mediated vascular dysfunction. In support of this, we found an association between increasing concentrations of plasma apelin and ELA, and big ET‐1 as well as big ET‐1:ET‐1 ratio. As ET‐1 binds with high affinity to its receptors and is rapidly cleared, plasma concentrations may not accurately reflect its production. Measurement of its precursor, big ET‐1, and the ratio of big ET‐1 to ET‐1, provide estimates of peptide production.^29^, ^30^ Given apelin is also produced by endothelial cells, our findings might suggest a simultaneous increase in apelin production as a compensatory mechanism for increased ET‐1 production. Our group has previously shown apelin infusion increases forearm blood flow in patients with heart failure.^12^ It would be of interest if apelin similarly improved endothelial function in CKD. A new treatment that not only improves renal but also cardiovascular measures on top of standard care would be particularly attractive for this at‐risk group of patients.

Baseline plasma apelin concentration associated independently with eGFR decline. For each 1 pg/mL increase in plasma apelin we find the odds of reaching a 50% decline in eGFR at 5 years increased ~9‐fold. Patients who reached ESKD by 5 years also had significantly higher baseline plasma apelin levels. The ability of a circulating cardiovascular peptide to predict poorer patient outcomes has been shown before. For example, plasma ET‐1 predicts mortality following myocardial infarction and adverse outcomes in patients with heart failure.^52^ In a cohort of haemodialysis patients, higher baseline ET‐1 level associates with increased mortality and hospitalization.^53^ Unexpectedly, we found no association between baseline eGFR and eGFR decline. This probably reflects the fact that 60% of our study cohort had a baseline GFR of >60 mL/min/1.73m^2^.

We recognize the limitations of our study. We present associations of plasma apelin and ELA concentrations and causation cannot be determined here. With respect to the increase in plasma apelin concentrations in CKD, we cannot discriminate between impaired renal clearance and increased vascular production. Nevertheless, apelin is an attractive, new potential therapy for CKD and our findings suggest that clinical trials are now urgently needed in this space.

## COMPETING INTERESTS

D.E.N. reports grants and personal fees from Bristol‐Myers Squibb and personal fees from Amgen, outside the submitted work.

## CONTRIBUTORS

N.D., A.P.D., J.J.M. and D.E.N. were responsible for the overall study design. Immunohistochemistry & plasma apelin/ELA measurements: D.N., T.L.W. and R.E.K. designed and carried out experiments and data analysis and A.P.D. and J.J.M. designed and supervised experiments, performed data analysis, and contributed grant support and facilities. Clinical studies: N.D. performed the original clinical studies. F.A.C. and P.J.G. carried out the data analysis. F.A.C. wrote the manuscript and all authors contributed to its revision.

## Supporting information


**TABLE S1** Multivariable analysis showing no independent associations with plasma ELA. *PWV*: pulse wave velocity; *ET‐1:* endothelin‐1; *ADMA:* asymmetric dimethylarginine; *IL‐6:* interleukin 6.
**TABLE S2** Primer sequences or IDs for RT‐qPCR experiments.
**TABLE S3** List of antibodies showing the company, immunogen, concentration used and species.
**FIGURE S1** Expression of *APLN*, *APELA* and *APLNR* mRNA in human kidney. (A) Whole kidney; (B) separated into cortex and medulla. No statistically significant differences between *APLN*, *APELA* or *APLNR* expression were present. (C) Whole kidney concentrations of apelin and ELA were similar although reached statistical significance. Data represent mean ± SEM.
**FIGURE S2** Expression of apelin (E–H), ELA (I–M) and apelin receptor (N–Q) protein in renal blood vessels. Apelin, ELA and apelin receptor immunoreactivity colocalised with von Willebrand Factor (vWF), an endothelial marker. Negative (−ve) control (A); primary antibody omitted. Scale bar = 50 μm.
**FIGURE S3** (A) Paraffin‐embedded cortical sections were used to determine the location of the apelin system within the proximal convoluted tubule and/or descending loop of Henle. Apelin appeared to be expressed mainly in the apical membrane (A–D) while ELA and the apelin receptor were expressed on both the apical and basolateral membranes (E–L). (B) In sections of human kidney, these are examples of negative controls in which the primary antibodies for apelin receptor (B) and apelin peptide (E; seen in figure in manuscript and S2 and S3A above) are omitted and only the secondary antibodies (green 488 nm [H] and red 555 nm [K] are included. A, D, G, J show Hoechst nuclear staining and C, F, I and L show the corresponding overlays. Some autofluorescence is visible associated with the elastic lamina of blood vessels (H).
**FIGURE S4** Differences in plasma apelin (A) and ELA (Ela; B) concentrations in men and women. CKD: chronic kidney disease. We included 128 patients with CKD and 27 healthy volunteers.
**FIGURE S5** Associations of plasma apelin concentration (A), plasma ELA concentration (B), and kidney function (C) with the bigET‐1:ET‐1 ratio. eGFR: estimated glomerular filtration rate; bigET‐1: big endothelin‐1; ET‐1: endothelin‐1. We included 128 patients with CKD and 27 healthy volunteers.

## Data Availability

The data underlying this study will be made available upon reasonable request to the corresponding author (N.D.).
