# Supplementary material for: Apelin is expressed throughout the human kidney, is elevated in chronic kidney disease & associates independently with decline in kidney function
Source: Br J Clin Pharmacol. 2022 Jul 19;88(12):5295–306. doi: 10.1111/bcp.15446 (PMC9796317; doi:10.1111/bcp.15446)
Supplement: Supplementary file 1 — TABLE S1 Multivariable analysis showing no independent associations with plasma ELA. PWV: pulse wave velocity; ET‐1: endothelin‐1; ADMA: asymmetric dimethylarginine; IL‐6: interleukin 6. TABLE S2 Primer sequences or IDs for RT‐qPCR experiments. TABLE S3 List of antibodies showing the company, immunogen, concentration used and species. FIGURE S1 Expression of APLN, APELA and APLNR mRNA in human kidney. (A) Whole kidney; (B) separated into cortex and medulla. No statistically significant differences between APLN, APELA or APLNR expression were present. (C) Whole kidney concentrations of apelin and ELA were similar although reached statistical significance. Data represent mean ± SEM. FIGURE S2 Expression of apelin (E–H), ELA (I–M) and apelin receptor (N–Q) protein in renal blood vessels. Apelin, ELA and apelin receptor immunoreactivity colocalised with von Willebrand Factor (vWF), an endothelial marker. Negative (−ve) control (A); primary antibody omitted. Scale bar = 50 μm. FIGURE S3 (A) Paraffin‐embedded cortical sections were used to determine the location of the apelin system within the proximal convoluted tubule and/or descending loop of Henle. Apelin appeared to be expressed mainly in the apical membrane (A–D) while ELA and the apelin receptor were expressed on both the apical and basolateral membranes (E–L). (B) In sections of human kidney, these are examples of negative controls in which the primary antibodies for apelin receptor (B) and apelin peptide (E; seen in figure in manuscript and S2 and S3A above) are omitted and only the secondary antibodies (green 488 nm [H] and red 555 nm [K] are included. A, D, G, J show Hoechst nuclear staining and C, F, I and L show the corresponding overlays. Some autofluorescence is visible associated with the elastic lamina of blood vessels (H). FIGURE S4 Differences in plasma apelin (A) and ELA (Ela; B) concentrations in men and women. CKD: chronic kidney disease. We included 128 patients with CKD and 27 healthy volunteers. F [file BCP-88-5295-s001.docx]

**DATA SUPPLEMENT**

**Apelin is expressed throughout the human kidney, is elevated in chronic kidney disease & associates independently with decline in kidney function**

**Methods**

***Tissue studies***

*Human tissue samples*

Histologically normal human kidney was collected with informed consent and ethical approval (05/Q104/142), from patients with kidney cancer undergoing surgery. For some assays, samples were dissected into renal cortex and medulla. Tissue was frozen and stored at -70^o^C until used.

*Total RNA extraction*

Total RNA was extracted from ~200 mg of human kidney (whole kidney, n=5; cortex and medulla, n=6 each) using the PureLink RNA Mini Kit (RNA Ambion, Life Technologies) according to manufacturer instructions. Briefly, each kidney sample was chopped into tiny pieces with a sterile blade and transferred to a lysing matrix D tube (MP Biomedicals, USA) with 1 ml TRIzol. Tissues were homogenized using the FastPrep-24TM 5G system (MP Biomedicals, USA) for up to three runs at 6.5 m/s for 45 sec, and incubated (5 min, room temperature (RT)) with chloroform (200 µl). Homogenates were mixed by vigorous shaking (15 sec), incubated (3 min, RT) and centrifuged (12,000 x*g*, 15 min, 4°C). The resulting supernatant was then transferred to fresh tubes, mixed with an equal volume of freshly prepared 70% ethanol and transferred to a spin cartridge. The cartridge was spun (12,000 x*g*, 15 sec, RT) to remove TRIzol and chloroform. Genomic DNA was digested by incubating with PureLink DNAse mixture (containing 8 μl 10x DNAse I reaction buffer, 10 μl DNAse (~3 U/μl) and 62 μl DNAse-free water) for 15 min. After three more washes, the spin column was dried to evaporate residual ethanol by centrifugation (12,000 x*g*, 1 min, RT) and final RNA eluted. Extracted RNA was quantifying RNA yield using NanoDrop 1000 spectrophotometer (Wilmington, USA), and stored at -70°C until needed.

*Synthesis and purification of cDNA*

## First-strand cDNA was synthesized using the Promega Reverse Transcription System kit (A3500) according to manufacturer protocol. Briefly, total RNA (1 μg) was incubated with Oligo dT and Random primers (1 μl each) made up to 10.9 μl with RNase-free water and denatured at 70°C for 10 min before cooling at 4°C for 5 min on a ProFlex™ PCR System (Applied Biosystems). After adding the reverse transcription mix (comprising 4 μl MgCl_2_, 2 μl 10x reverse transcription buffer, 2 μl dNTP, 0.5 μl RNAsin ribonuclease inhibitor & 0.6 μl AMV Reverse Transcriptase), first-strand cDNA synthesis was performed under these conditions: 22°C for 5 min, 40°C for 1 h, 95°C for 5 min and 4°C for 5 min. In some cases, the resulting cDNA was purified using the Promega Wizard SV Gel and PCR Clean-Up System kit (Cat. No.: A9282) according to manufacturer protocol.

***Quantitative real-time polymerase chain reaction (qPCR)***

cDNA (3.75 μl) was added to 11.25 μl of qPCR master mix (comprising Applied Biosystems TaqMan Gene Expression Master Mix; *APELA*, *APLN* or *APLNR* double-dye primer-probe, and water in the ratio of 10:1:4) per well of DNase-/RNAse-free MicroAmp 96-well plates on ice. The reaction was performed in triplicates for each gene. Human 18S rRNA (Life Technologies) was used as an internal control. The primer sequence or IDs are shown in **Table S2**. The expression of *APELA, APLN and APLNR,* were normalized to 18S using the comparative C_T_ method as previously described.^1,2^

***Kidney homogenate preparation***

For quantification of apelin and ELA peptide concentrations in kidney tissues, human kidney (~250 mg) samples (n=5) were transferred into Lysing MatrixD ceramic bead tubes and stored at -70 °C until needed. Boiling water (1 mL, 100 °C) was added to each tube as previously described.^3^ Samples were boiled for 20 mins, cooled to room temperature, homogenised using the FastPrep-24TM 5G system (MP Biomedicals, USA) for 3 runs (6.0 m/s, 40 secs) and centrifuged (1000 xg, 5 mins, 4 °C) to obtain extracted peptides in the supernatant. Resulting samples were stored at -70°C until required.

***Saturation binding experiment***

Protein homogenates were diluted to 4.5 mg/ml (1.5 mg/ml final concentration) in assay buffer (Tris 50 mM, MgCl_2_ 5 nM, pH 7.4). A two-fold serial dilution of [^125^I]apelin-13 was performed in assay buffer to achieve a final concentration range of 2 nM - 3.9 pM in sigmacoted tubes. Protein samples (100 µl) were incubated with increasing concentrations of [^125^I]apelin-13 (100 µl) in the absence (total binding) or presence of 2 μM [Pyr^1^]apelin-13 (nonspecific binding), for 90 min at 4°C, until equilibrium was reached and centrifuged (20,000 x*g*, 10 min, 4°C) to terminate the reaction. The resulting pellets were washed with 500 µl ice-cold wash buffer (Tris-HCl buffer; 50 mM Tris, pH 7.4) before second centrifugation at the same settings. Pellets and solutions of total and nonspecific binding were counted on a Cobra II Auto-Gamma radiation counter (Packard) to obtain radioactivity in disintegrations per minute (DPM). This was used in the KELL program to determine the concentration of [^125^I]apelin-13 that bind 50% of the apelin receptors (K_D_, the equilibrium dissociation constant) and apelin receptor density (B_max_).^4^ Saturation binding data analyses were performed using the non-linear iterative curve-fitting program, RADLIG software (version 6) of the Equilibrium Binding Data Analysis (EBDA) and LIGAND programs (KELL, BIOSOFT, Cambridge, UK).^4^ This program performs an initial analysis of data obtained from saturation studies by converting radioactivity in disintegrations per minute (DPM) to concentrations using the specific activity of the radiolabel (~2200 Ci/mmol for [^125^I]apelin-13), where 1 Ci = 2.22x10^12^ DPM. This conversion factor was used to convert specific activity of the radiolabel to DPM/fmol (4.884x10^3^ DPM/fmol), which was further converted to specific binding (fmol/mg protein) using the known protein concentration used in the assay. The Scatchard and Hill analyses performed by EBDA provided initial estimates that were used in the LIGAND component of the KELL Program.^5^ This component of the program uses weighted, non-linear iterative curve-fitting to derive the final values for receptor density (B_Max_) and KD. The (B_Max_) and K_D_ were then expressed as mean±SEM.

*Receptor autoradiography*

Human (n=6) sections were pre-incubated for 20 mins in binding buffer (50 mM Tris, 5 mM MgCl_2_, pH 7.4) at room temperature before incubating for 90 mins with 0.5 nM [^125^I]apelin-13 in binding buffer. Nonspecific binding was defined as binding of [^125^I]apelin-13 (0.5 nM) in the presence of 2 µM [Pyr^1^]apelin-13. Slides were then washed (3x 5 mins) with ice-cold Tris-HCl buffer (50 mM Tris, pH 7.4), before rinsing in ice-cold deionized water. All slides were air-dried at room temperature before being exposed to emulsion-coated film (Kodak, USA) for 1-2 weeks at room temperature in the dark. Films were developed in 20% v/v developer for 3-5 min with rocking, rinsed in deionised water for 1 min before incubating for 10 min in 20% v/v fixer with constant agitation in the dark. The film was washed in running water for 15 mins, air-dried and imaged using a PixeLINK Camera attached a Wild M3Z microscope (Heerbrugg, Switzerland).

***Immunofluorescence double staining***

To precisely identify the cellular distribution of apelin, elabela and apelin receptor along the nephron in human kidney (n=4-5), dual-label immunofluorescence was performed on fresh-frozen human kidney sections using specific markers (**Table S3**). Sections were air-dried overnight and fixed in acetone for 10 min at RT. Slides were ringed with a hydrophobic pen and incubated with 5% goat serum in 1x PBS for 2 hrs at room temperature to block nonspecific binding. Primary antibodies were prepared in PBST (PBS and 0.01% Tween 20) containing 3% goat serum and 250 μl of PBST solution containing antibodies added to each slide. All antibodies used and their concentrations are shown in **Table S3**. Buffer (PBST containing 3% goat serum) instead of the primary antibody was added to negative control sections before all slides were incubated with antibodies overnight at 4^o^C. Following three 5 min washes in ice-cold PBST, secondary antibodies (Alexa Fluor 488 goat anti-rabbit IgG and Alexa Fluor 568 goat anti-mouse IgG) and Hoechst (1:100) prepared in buffer were added and incubated for 1 h at RT. Sections were washed 3x 5 min in ice-cold PBST and mounted with ProLong Gold Antifade reagent. Slides were imaged using Zeiss 510 Meta confocal laser scanning microscope (Heidelberg, Germany) or Leica TCS SP8 confocal laser scanning microscope (Leica Microsystems, Milton Keynes, UK) at x40 magnification. Images were processed using Fiji (ImageJ) for background subtraction using the rolling ball method before merging channels. Additional automated images (16 bit, 0.325 x 0.325 μm scaling per pixel) were obtained using a Slide Scanner Axio Scan.Z1 (Carl Zeiss Microscopy GmbH, Gottingen, Germany) microscope with a Plan-Apochromat 20x/NA0.8 M27 objective lens connected to a Hamamatsu Orca Flash camera. Acquired images were visualised using Orbit Image Analysis Software (v3.65).

***Clinical studies***

*Participants*

Patients with non-diabetic CKD were recruited from the general renal outpatient clinic at the Royal Infirmary of Edinburgh. Patients were excluded if they had known cardiovascular disease (ischemic heart disease, congestive cardiac failure or stroke) to avoid a confounding effect on the apelin system.^6^ Healthy volunteers were recruited from established local volunteer databases. Ethical approval was obtained for all studies in accordance with the Declaration of Helsinki, and all subjects provided written informed consent.

*Renal function*

Glomerular filtration rate was estimated using the Chronic Kidney Disease Epidemiology Collaboration (CKD-EPI) equation (eGFR) in 121 participants (including all healthy volunteers) and measured precisely using inulin clearance (iGFR) in the remaining 34 participants.^7^ Urine albumin was measured using a colorimetric method with pyrogallol red and urine sodium using an ion selective electrode.^8,9^ CKD was defined as an eGFR/iGFR <60 mL/min/1.73m^2^.

*Cardiovascular assessment*

Subjects rested supine for 15 min prior to cardiovascular assessments. Systolic and diastolic BP were recorded using an appropriately sized cuff and a validated oscillometric sphygmomanometer, the Omron HEM-705CP.^10^ Values were the average of two consecutive readings that were within 10 mmHg of each other. Mean arterial pressure was calculated as the sum of diastolic BP and a third of the pulse pressure.

Carotid-femoral pulse wave velocity was measured by the foot-to-foot wave velocity method, using the SphygmoCor system (SphygmoCor Mx version 6.31, AtCor Medical).^11^ Pulse wave velocity was taken as the average of two readings within 10% of each other.

Brachial artery flow-mediated dilation was used to assess endothelium-dependent vasomotor function as described previously.^12^ Ultrasound measurements were taken at the brachial artery, distal to the blood pressure cuff. Flow-mediated dilation was quantified both as the peak change from baseline and as the area under the curve of the change from baseline in brachial artery diameter after 5 min of forearm ischaemia.

*Biochemical analyses*

Venous blood samples were collected in ethylene diamine tetraacetic acid (EDTA) and gel tubes and centrifuged immediately (2500 x *g* for 20 min for EDTA, 3000 x *g* for 15 min for gel, both at 4ºC). Samples were stored at -80ºC until analysis. Plasma endothelin-1 (ET-1) and big ET-1 concentrations were determined by radioimmunoassay (Peninsular Laboratories, UK; limits of detection 0.25 pg/mL and 1.0 pg/mL, respectively; intra- and inter-assay coefficients of variation 6.3% and 7.2% and 6.3% and 7.5%, respectively).^13^ Plasma asymmetric dimethylarginine (ADMA) concentration was measured using an optimised fully validated high-performance liquid chromatography method (limit of detection 0.001 μmol/L; assay variations 1.9% and 2.3%).^14^ Plasma interleukin-6 (IL-6) concentration was quantified by an enzyme-linked immunosorbent assay (Cayman Chemical, US; limit of detection 0.7 pg/mL; assay variations 5% and 15%, respectively). High sensitivity C-reactive protein (CRP) concentration was quantified in the hospital laboratory using a latex particle enhanced immunoturbidimetry technique (Vitros® 5, 1 FS Chemistry Systems, Ortho-Clinical Diagnostics, Inc., New York, USA) (limit of detection 0.2 mg/L; assay variations 2.3% and 5%).

*Measurement of plasma apelin and elabela plasma concentrations*

Published plasma concentrations of apelin, and more recently elabela, vary widely and likely reflect differing sample preparation.^15^ There are several biologically active apelin peptide fragments however the predominant isoform in the cardiovascular system is the pyroglutamated apelin-13 ([Pyr^1^]apelin-13).^16^ We have recently developed a high-resolution liquid chromatography with tandem mass spectrometry method for detection and quantification of [Pyr^1^]apelin-13 in human plasma.^17,18^ We used this to validate the plasma apelin concentrations measured in this study using the commercially available enzyme-linked immunosorbent assay kit (EK-057-23 and EK-007-19, Phoenix Pharmaceuticals, Burlingame, USA). This ELISA was also used to measure the concentration of apelin and ELA in human kidney homogenates.

*Statistical analysis*

All data were tested for normality with log transformation as appropriate. Data are presented as median [interquartile interval] or mean±SEM where indicated. Comparisons were performed by Student’s *t*-test or Mann-Whitney test as appropriate, or one-way ANOVA if ≥3 groups. Correlations were performed using Pearson’s or Spearman’s tests. Multivariate linear and logistic regression was performed with variables shown to be significant on simple regression. Results were considered statistically significant if two-sided p <0.05. Statistical analyses were carried out on Prism version 8.3.1 or R version 3.5.1.

**Table S1.** Multivariable analysis showing no independent associations with plasma ELA. *PWV*: pulse wave velocity; *ET-1:* endothelin-1; *ADMA:* asymmetric dimethylarginine; *IL-6:* interleukin 6.

|  | Beta coefficient | Standard error | *t* statistic | *p* value |
| --- | --- | --- | --- | --- |
| Log PWV | 0.45 | 0.55 | 0.82 | 0.42 |
| ET-1 | 0.03 | 0.07 | 0.44 | 0.66 |
| ADMA | 0.89 | 0.59 | 1.49 | 0.14 |
| Log IL-6 | 0.34 | 0.18 | 1.88 | 0.07 |

**Table S2.** Primer sequences or IDs for RT-qPCR experiments

| Target gene | Primer sequence or ID |
| --- | --- |
| Human *APELA* (Primer design) | Sense GAAGAAGAAGAGGAGTGAAGGA Antisense CCATTCCAGGTGCTTTCAAAT |
| *Primers from ThermoFisher* | |
| Human 18S rRNA | Hs99999901_s1 |
| Human *APLN* | Hs00175572_m1 |
| Human *APLNR* | Hs00270873_s1 |

**Table S3.** List of antibodies showing the company, immunogen, concentration used and species.

| Protein (human) | Species | Immunogen | Concentration used | Company |
| --- | --- | --- | --- | --- |
| Primary antibodies | | | | |
| [pGlu^1^]ELA-32 | Rabbit | Full length [pGlu^1^]ELA-32 | 1:300 | Phoenix Pharmaceuticals |
| Apelin | Rabbit | C-terminal | 1:50 | Abcam  (Cat No.: ab59469) |
| Apelin receptor | Rabbit | C-terminal | 1:100 | Abcam  (Cat No.: ab84296) |
| Von-Willebrand factor (vWF) | Mouse | Unknown | 1:50 | Dako  (Cat. No.: M061601-2) |
| Aquaporin 1 (AQP1) | Mouse | Unknown | 1:100 | Santa Cruz  (Cat No.: sc-25287) |
| Aquaporin 4 (AQP4) | Mouse | Unknown | 1:100 | Abcam  (Cat No.: ab9512) |
| Sodium potassium chloride cotransporter | Mouse | Unknown | 1:100 | Santa Cruz  (Cat No.: sc-293222) |
| Transient receptor potential melastatin 6 | Mouse | Unknown | 1:100 | Santa Cruz  (Cat No.: sc-365536) |
| Sodium glucose cotransporter 2 (SGLT2) | Mouse | Unknown | 1:50 | Santa Cruz  (Cat No.: sc-393350) |
| Renin | Mouse | Recombinant Human Renin (full length) | 1:100 | Abcam (Cat. No: ab134783) |
| Table S3 continued | | | | |
| Protein (human) | Species | Immunogen | Concentration used | Company |
| Secondary antibodies | | | | |
| Alexa Fluor 488 goat anti-rabbit IgG | Goat | Rabbit IgG | 1:200 | Invitrogen  (Cat. No.: 11034) |
| Alexa Fluor 568 goat anti-mouse IgG | Goat | Mouse IgG | 1:200 | Invitrogen  (Cat No.: 11031) |

**Figure S1.** Expression of *APLN*, *APELA* and *APLNR* mRNA in human kidney. A) Whole kidney; B) separated into cortex and medulla. No statistically significant differences between *APLN*, *APELA* or *APLNR* expression were present. C) Whole kidney concentrations of apelin and ELA were similar although reached statistical significance. Data represent mean±SEM.

**
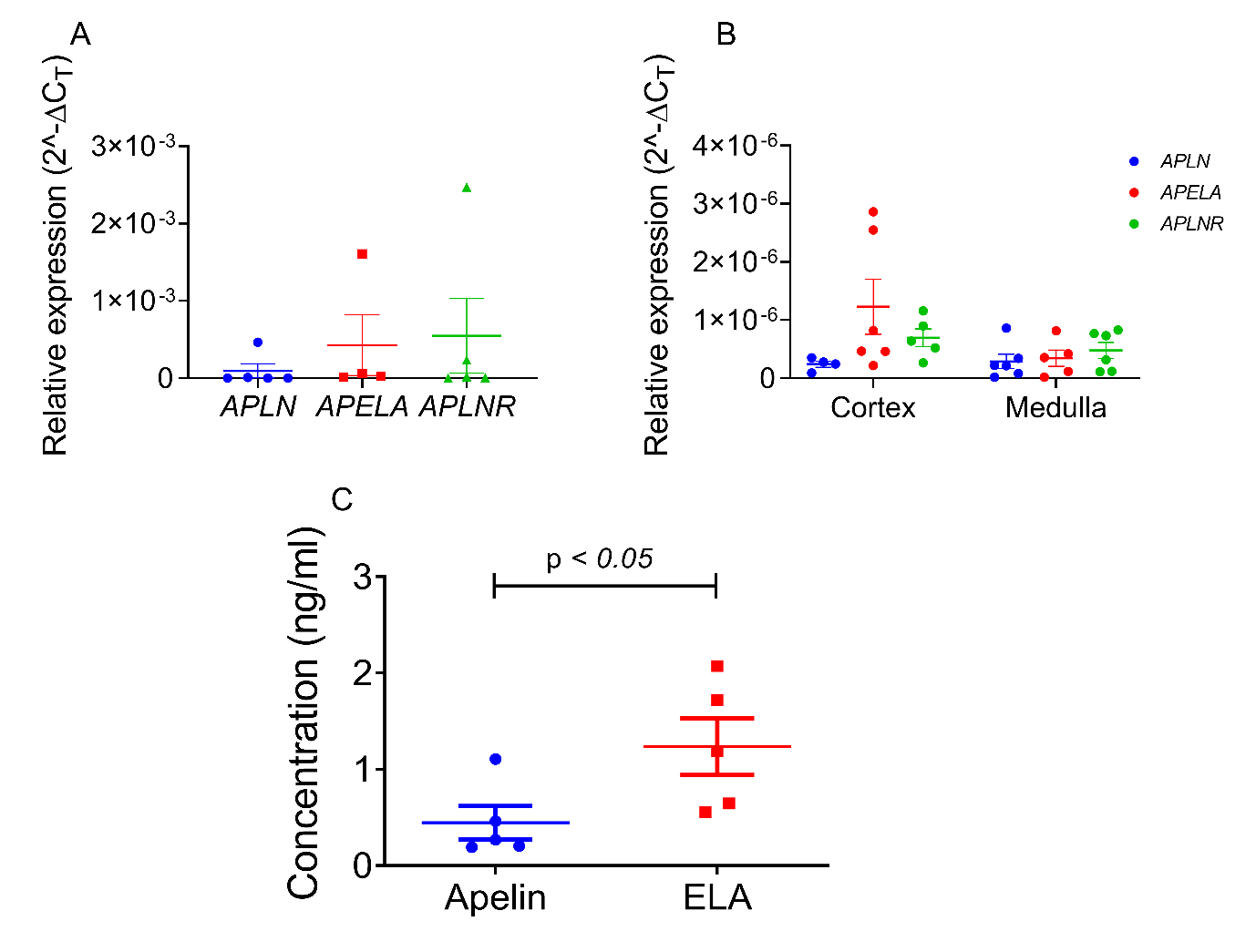
**

**Figure S2.** Expression of apelin (E-H), ELA (I-M) and apelin receptor (N-Q) protein in renal blood vessels. Apelin, ELA and apelin receptor immunoreactivity colocalised with von Willebrand Factor (vWF), an endothelial marker. Negative (-ve) control (A) – primary antibody omitted. *Scale bar = 50µm.*


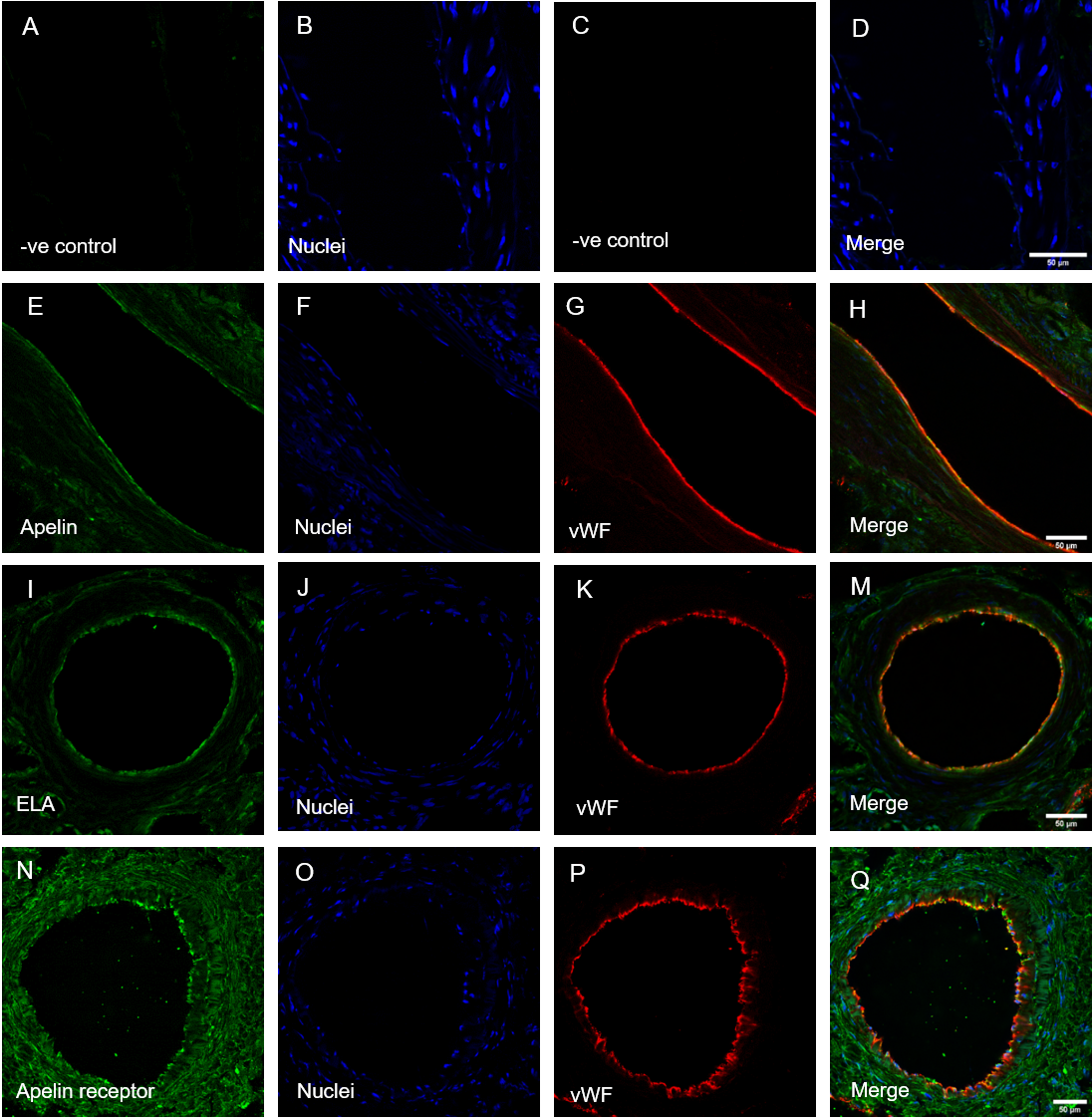


**Figure S3A.** Paraffin-embedded cortical sections were used to determine the location of the apelin system within the proximal convoluted tubule and/or descending loop of Henle. Apelin appeared to be expressed mainly in the apical membrane (A-D) while ELA and the apelin receptor were expressed on both the apical and basolateral membranes (E-L).


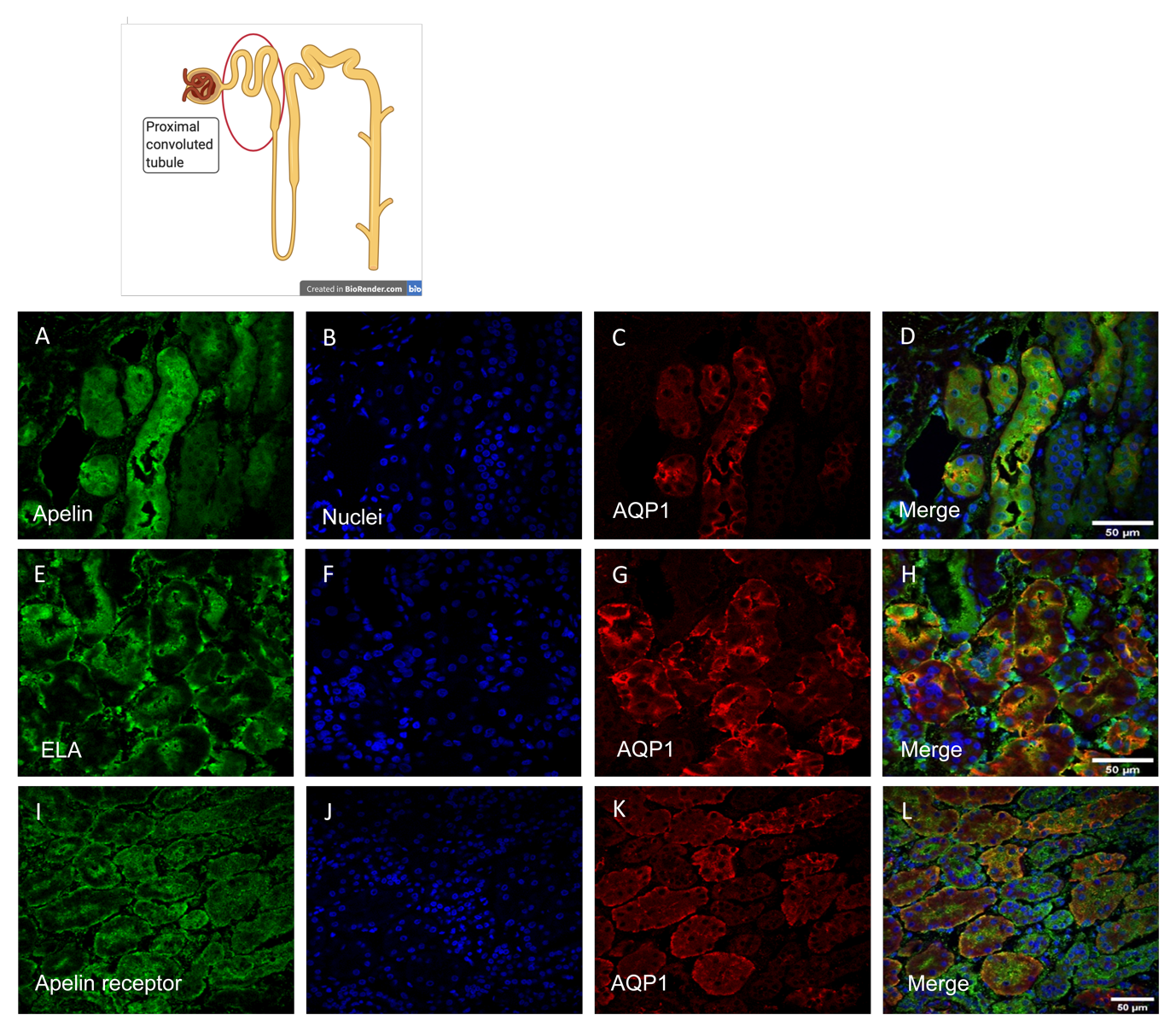


**Figure S3 B.** In sections of human kidney, these are examples of negative controls in which the primary antibodies for apelin receptor (B) and apelin peptide (E) (seen in figure in manuscript and S2 and S3A above) are omitted and only the secondary antibodies (green 488nm (H) and red 555nm (K) are included. A, D, G, J show Hoechst nuclear staining and C, F, I and L show the corresponding overlays. Some autofluorescence is visible associated with the elastic lamina of blood vessels (H).


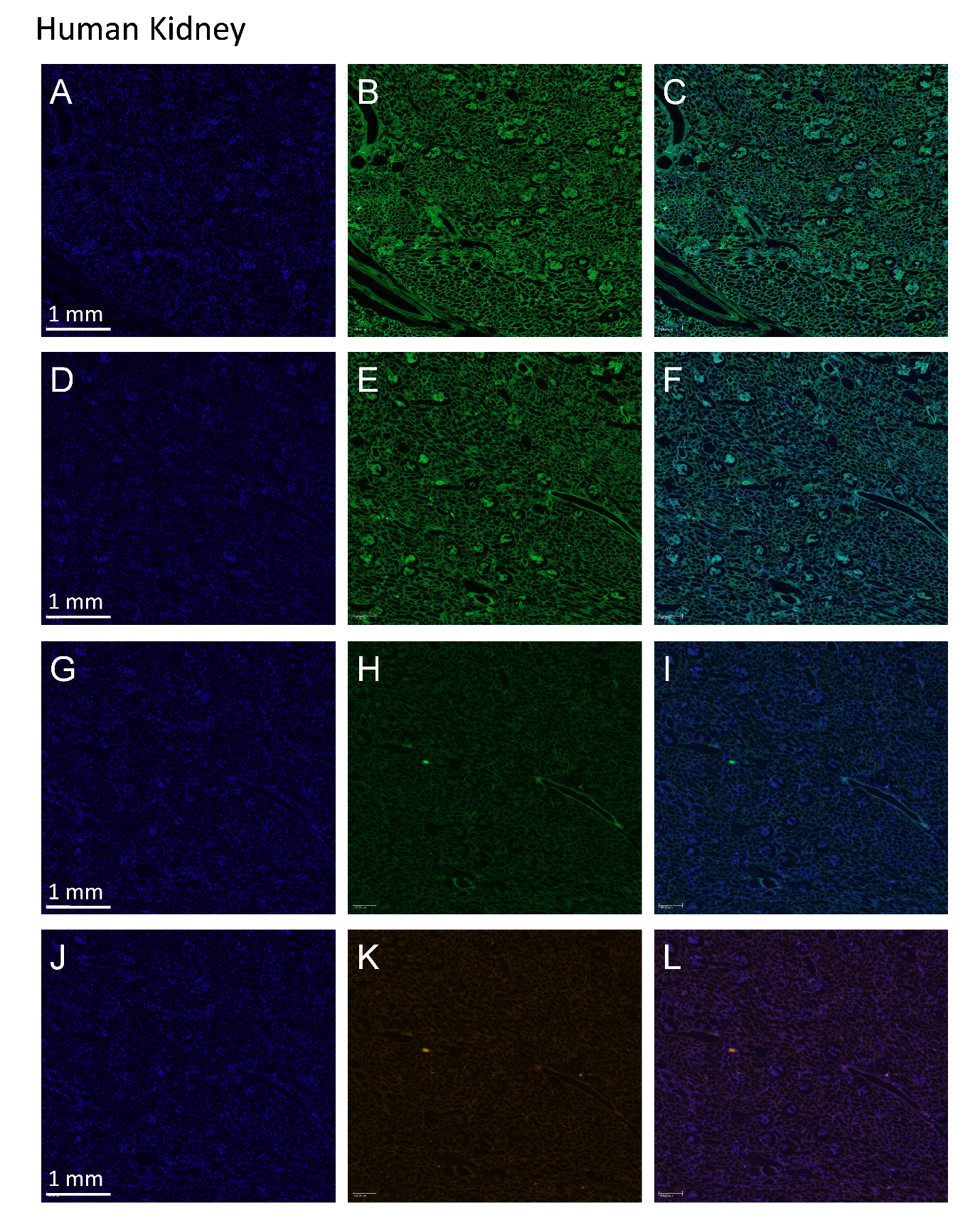


**Figure S4.** Differences in plasma apelin (A) and ELA (Ela) (B) concentrations in men and women. *CKD*: chronic kidney disease. We included 128 patients with CKD and 27 healthy volunteers.


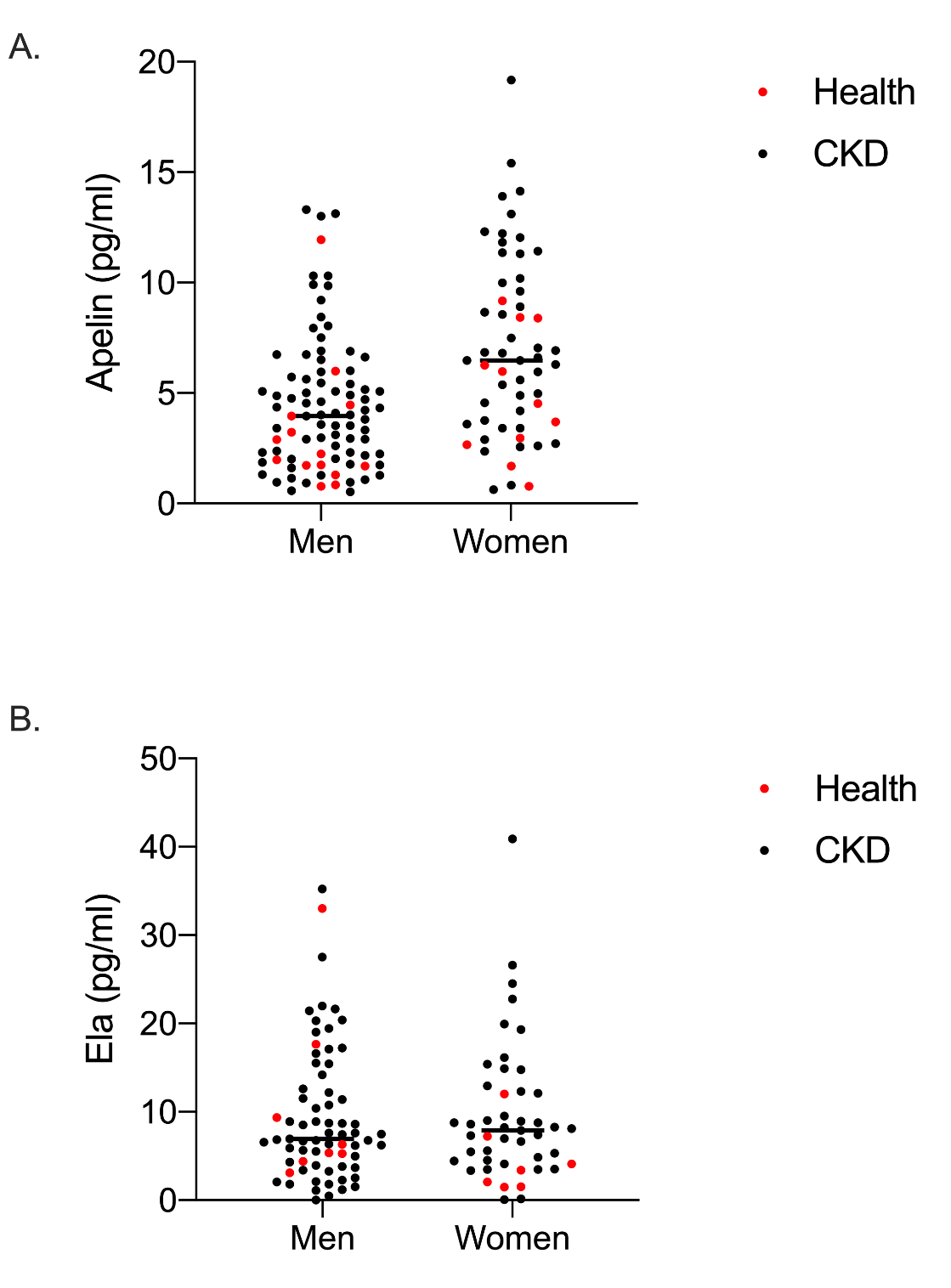


**Figure S5.** Associations of plasma apelin concentration (A), plasma ELA concentration (B), and kidney function (C) with the bigET-1:ET-1 ratio. *eGFR*: estimated glomerular filtration rate; *bigET-1*: big endothelin-1; *ET-1*: endothelin-1. We included 128 patients with CKD and 27 healthy volunteers.

**
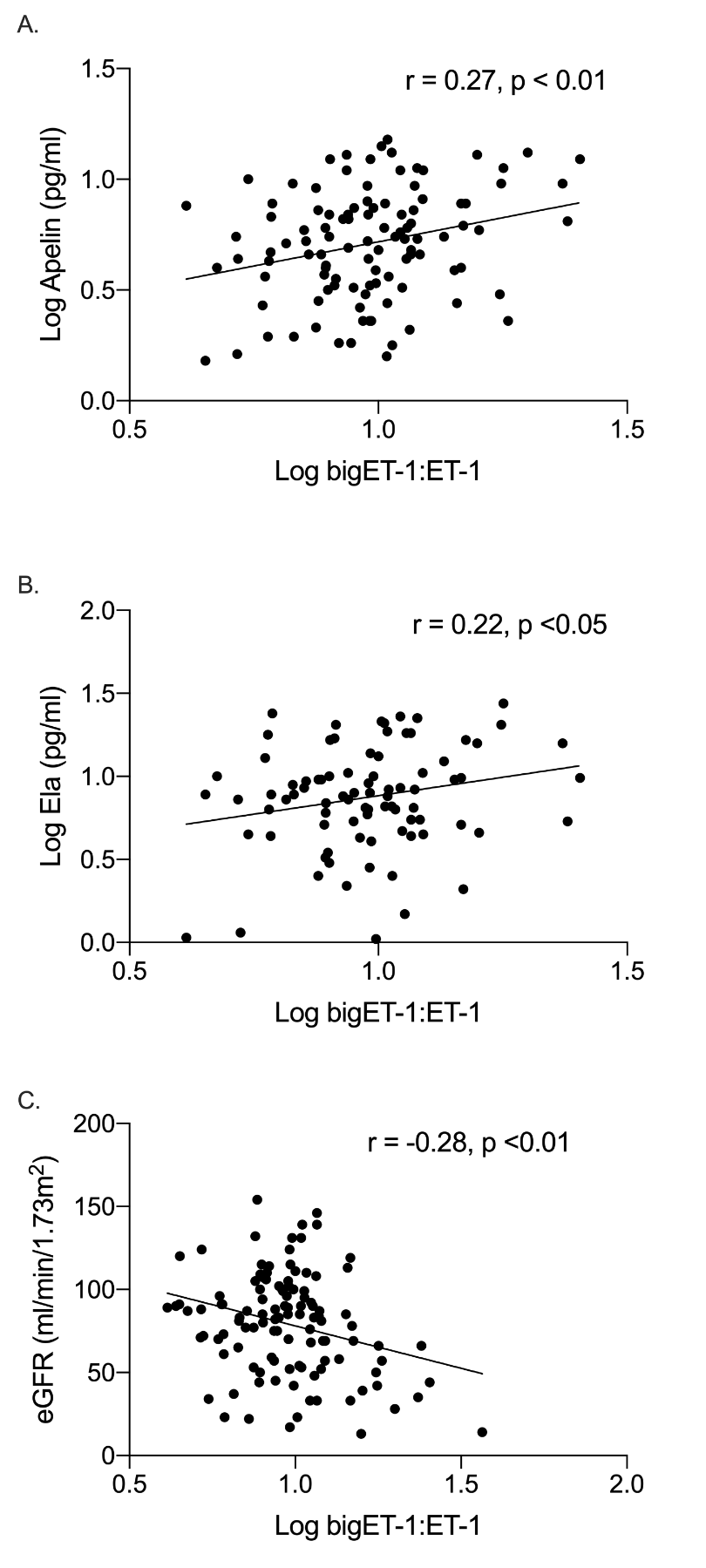
**

**References**

1. Livak KJ, Schmittgen TD. Analysis of relative gene expression data using real-time quantitative PCR and the 2(-Delta Delta C(T)) Method. *Methods.* 2001;25(4):402-408.

2. Schmittgen TD, Livak KJ. Analyzing real-time PCR data by the comparative C(T) method. *Nat Protoc.* 2008;3(6):1101-1108.

3. Pauli A, Norris ML, Valen E, et al. Toddler: an embryonic signal that promotes cell movement via Apelin receptors. *Science.* 2014;343(6172):1248636.

4. McPherson GA. Analysis of radioligand binding experiments: A collection of computer programs for the IBM PC. *Journal of Pharmacological Methods.* 1985;14(3):213-228.

5. Maguire JJ, Kuc RE, Davenport AP. Radioligand binding assays and their analysis. *Methods Mol Biol.* 2012;897:31-77.

6. Japp AG, Newby DE. The apelin-APJ system in heart failure: pathophysiologic relevance and therapeutic potential. *Biochem Pharmacol.* 2008;75(10):1882-1892.

7. Dhaun N, MacIntyre IM, Kerr D, et al. Selective endothelin-A receptor antagonism reduces proteinuria, blood pressure, and arterial stiffness in chronic proteinuric kidney disease. *Hypertension.* 2011;57(4):772-779.

8. Watanabe N, Kamei S, Ohkubo A, et al. Urinary protein as measured with a pyrogallol red-molybdate complex, manually and in a Hitachi 726 automated analyzer. *Clin Chem.* 1986;32(8):1551-1554.

9. Pelleg A, Levy GB. Determination of Na+ and K+ in urine with ion-selective electrodes in an automated analyzer. *Clin Chem.* 1975;21(11):1572-1574.

10. O’Brien E MF, Atkins N, Thomas M. Evaluation of three devices for self-measurement of b lood pressure according to the revised British Hypertension Society Protocol: the Omron HEM-705CP, Philips HP5332, and Nissei DS-175. *Blood Press Monit.* 1996;1:55-61.

11. Oliver JJ, Webb DJ. Noninvasive assessment of arterial stiffness and risk of atherosclerotic events. *Arterioscler Thromb Vasc Biol.* 2003;23(4):554-566.

12. Corretti MC, Anderson TJ, Benjamin EJ, et al. Guidelines for the ultrasound assessment of endothelial-dependent flow-mediated vasodilation of the brachial artery. *Journal of the American College of Cardiology.* 2002;39(2):257-265.

13. Strachan FE, Spratt JC, Wilkinson IB, Johnston NR, Gray GA, Webb DJ. Systemic blockade of the endothelin-B receptor increases peripheral vascular resistance in healthy men. *Hypertension.* 1999;33(1 Pt 2):581-585.

14. Blackwell S, O'Reilly DS, Talwar D. Biological variation of asymmetric dimethylarginine and related arginine metabolites and analytical performance goals for their measurement in human plasma. *Eur J Clin Invest.* 2007;37(5):364-371.

15. Georgiadou D, Boussata S, van Dijk M. ELABELA measurements by commercial ELISA kits require sample extraction. *Am J Physiol Endocrinol Metab.* 2019;317(6):E1218-E1219.

16. Maguire JJ, Kleinz MJ, Pitkin SL, Davenport AP. [Pyr1]apelin-13 identified as the predominant apelin isoform in the human heart: vasoactive mechanisms and inotropic action in disease. *Hypertension.* 2009;54(3):598-604.

17. Kay RG, Galvin S, Larraufie P, Reimann F, Gribble FM. Liquid chromatography/mass spectrometry based detection and semi-quantitative analysis of INSL5 in human and murine tissues. *Rapid Commun Mass Spectrom.* 2017;31(23):1963-1973.

18. Nyimanu D, Kay RG, Sulentic P, et al. Development and validation of an LC-MS/MS method for detection and quantification of in vivo derived metabolites of [Pyr(1)]apelin-13 in humans. *Sci Rep.* 2019;9(1):19934.
